# A time-resolved fluorescence microsphere-lateral flow immunochromatographic strip for quantitative detection of Pregnanediol-3-glucuronide in urine samples

**DOI:** 10.3389/fbioe.2023.1308725

**Published:** 2023-12-19

**Authors:** Jiasheng Lin, Sanhua Li, Benchen Ye, Weigang Zheng, Huihui Wang, Ying Liu, Dong Wang, Zaihui Wu, Wen-Fei Dong, Minghui Zan

**Affiliations:** ^1^ CAS Key Laboratory of Biomedical Diagnostics, Suzhou Institute of Biomedical Engineering and Technology, Chinese Academy of Science (CAS), Suzhou, China; ^2^ Zhengzhou Institute of Biomedical Engineering and Technology, Zhengzhou, China; ^3^ Henan Province Joint International Laboratory for Bioconjugation and Antibody Coupling, Zhengzhou, China; ^4^ Zhongke Technology Achievement Transfer and Transformation Center of Henan Province, Zhengzhou, China

**Keywords:** time-resolved fluorescence microspheres, Pregnanediol-3-glucuronide, lateral flow immunochromatographic, urine sample, competitive model

## Abstract

**Introduction:** Pregnanediol-3-glucuronide (PdG), as the main metabolite of progesterone in urine, plays a significant role in the prediction of ovulation, threatened abortion, and menstrual cycle maintenance.

**Methods:** To achieve a rapid and sensitive assay, we have designed a competitive model-based time-resolved fluorescence microsphere-lateral flow immunochromatography (TRFM-LFIA) strip.

**Results:** The optimized TRFM-LFIA strip exhibited a wonderful response to PdG over the range of 30–2,000 ng/mL, the corresponding limit of detection (LOD) was calculated as low as 8.39 ng/mL. More importantly, the TRFM-LFIA strip was innovatively used for the quantitative detection of PdG in urine sample, and excellent recovery results were also obtained, ranging from 97.39% to 112.64%.

**Discussion:** The TRFMLFIA strip possessed robust sensitivity and selectivity in the determination of PdG, indicating the great potential of being powerful tools in the biomedical and diagnosis region.

## 1 Introduction

Progesterone (P4) is a bioactive steroid hormone secreted by the corpus luteum in the ovaries (Cao et al., 2020), which plays a diagnostic role in ovulation prediction, threatened abortion, and menstrual cycle maintenance (Disha Kumari et al., 2022; Sun et al., 2023). Serum P4 concentrations increased specifically during the luteal phase after ovulation and are widely used to confirm ovulation in non-pregnant humans (Handelsman et al., 2021). Serum progesterone (P) levels are usually <1.5 ng/mL (4.77 nmol/L) during the follicular phase. After ovulation, there is a rapid increase in P. Minimum *p* values to confirm ovulation and luteinization have been variously cited at 1.8–5.0 ng/mL (5.72–15.90 nmol/L) (Usala, et al., 2021). However, this requires venipuncture during the luteal period and needs professionals which is not suitable for rapid self-testing at home. Pregnanediol-3-glucuronide (PdG) is the phase II (glucuronidated) excretory metabolite of pregnanediol (Handelsman, et al., 2021), the major urine metabolite of P4, whose concentration is positively correlated with the concentration of P4 in serum. Some PdG test strips were based on the threshold concept and set 5 μg/mL as the detection threshold to determine ovulation et al., 2019). The time distribution of serum P4 and urine PDG both peaked after ovulation (Munro et al., 1991; O'Connor et al., 2003). As a non-invasive ovulation test, the detection of PdG plays an important role in women planning pregnancy and implementing contraception.

Up to now, some methods have been reported about the detection of PdG in urine have been reported in the literature, including radioimmunoassay (RIA) (MacLean et al., 1981; Mendizabal et al., 1984; Baird et al., 1995), liquid chromatography-mass spectrometry (LC-MS) (Handelsman, et al., 2021), mass spectrometry (MS) (Moneti et al., 1985), enzyme-linked immunosorbent assay (ELISA) (Fabres et al., 1993; O'Connor, et al., 2003). However, most require professional technicians and expensive instruments, which limits their application in rapid detection. Therefore, it is urgent to develop a sensitive and economic assay for the detection of PdG in urine samples.

Immunochromatography has attracted more and more attention because of its simple operation, low cost, and time-saving, and some reports introduced the application of PdG immunochromatography strip based on colloidal gold technology (Leiva et al., 2019). The sensitivity of fluorescence immunochromatography is significantly higher than that of colloidal gold immunochromatography (Xie et al., 2014), which is more suitable for the quantitative detection of low concentration targets. Time-resolved fluorescence microsphere-lateral flow immunochromatography (TRFM-LFIA) is a novel immunoassay technique proposed in the 1980s (Soini and Kojola, 1983). TRFM is usually obtained by encapsulating lanthanide complexes in polystyrene materials or silica nanoparticles (Tang et al., 2017; Wang et al., 2023a). Compared with other fluorescent materials, TRFM possesses a longer fluorescence lifetime and a larger Stokes shift, which is conducive to reducing the influence of background fluorescence and improving the sensitivity of detection through time-resolved technology (Wang et al., 2017; Lei et al., 2019). In addition, the lanthanide complex is encapsulated inside the fluorescent microspheres to improve the stability of TRFM and thus the overall stability of the TRFM-LFIA strip. Coincidentally, the surface of the polystyrene microspheres modified with various functional groups was utilized to improve the coupling efficiency of proteins (antigens or antibodies) (Li et al., 2021a; Jiang et al., 2022). Therefore, TRFM-LFIA has great potential application in medical diagnosis and food safety analysis (Wang et al., 2023a; Xu et al., 2023).

In this study, we have designed a competitive model-based TRFM-LFIA strip for the detection of PdG in urine. The preparation parameters of the anti-PdG-mAb labeled with the TRFM (TRFM-mAb) probe and the TRFM-LFIA strip were determined respectively, and the performance of the TRFM-LFIA strip was evaluated by the sensitivity, stability, specificity, precision, and recovery. The TRFM-LFIA strip was applied to the evaluation of actual urine samples, and the rapid and sensitive quantitative detection of PdG in urine was realized. Therefore, the developed TRFM-LFIA strip exhibited enormous potential in various scenarios for the detection of PdG in urine samples and achieved accurate prediction of the ovulation period.

## 2 Materials and methods

### 2.1 Chemicals and apparatus

Chemicals and apparatus information can be found in Supplementary Material.

### 2.2 Preparation of TRFM-mAb

The carboxyl group on the surface of rare earth europium complex doped polystyrene fluorescent microspheres was connected to the amino group of antibodies by the chemical coupling method to form a stable amide bond (Zhang et al., 2017). Initially, 20 μL of TRFM (1% solids) was mixed with 100 μL MES solution (pH 6.0, 0.05 M) and underwent ultrasound for 1 min. After centrifugation at 15,000 *g* for 10 min, the supernatant was discarded and then repeat the above steps to wash TRFM three times. Subsequently, TRFM was added to 60 μL of MES solution (pH 6.0, 0.05 M), and 20 μL freshly prepared EDC solution (20 mg/mL) and 20 μL NHS solution (20 mg/mL) were added to incubate for 30 min at room temperature. The incubated microspheres were purified by centrifugation at 15,000 *g* and 8°C for 10 min and were washed twice with 100 μL of phosphate buffered saline (PBS) (pH 7.4, 0.01 M). A series of different volumes of anti-PdG-mAb (5.0 mg/mL) were added to optimize the antibody dosage. The reaction solution was continuously stirred at room temperature for 4 h. The 10% BSA blocking solution was slowly added into the tube, and the reaction was carried out at room temperature for 1 h. Finally, the TRFM-mAb conjugates were centrifugally washed with PBS solution and resuspension solution (PBS solution (pH 7.2, 0.1 M) containing 10% (w/v) sucrose, 5% (w/v) trehalose, 2% (v/v) Tween-20% and 0.5% (w/v) BSA), respectively, and then were resuspended in 200 μL of resuspension solution. The solution was stored at 4°C in darkness for future use.

The surface morphology, particle size, fluorescence property and surface group of the TRFM-mAb were characterized and analyzed.

### 2.3 Preparation of the TRFM-LFIA strip

**SCHEME 1:**
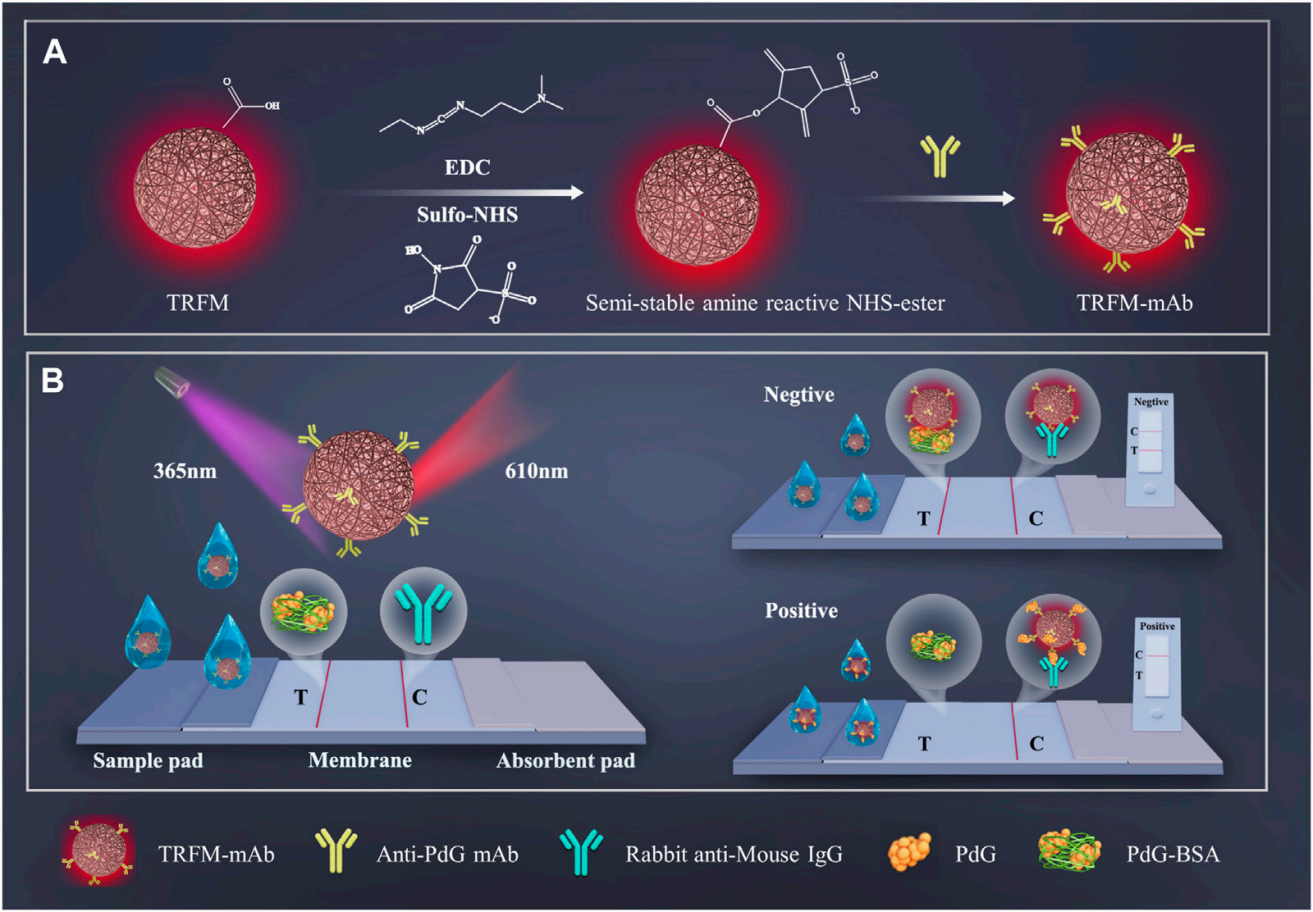
The schematic illustration of preparation process of TRFM-mAb **(A)** and quantitative detection process of PdG by TRFM-LFIA strip **(B)**.

As shown in Scheme 1, the TRFM-LFIA strip is mainly composed of and polyvinyl chloride (PVC) pad, sample pad, nitrocellulose (NC) membrane, and absorbent pad (Wang, et al., 2023b). The 0.2 mg/mL PdG-BSA conjugate solution and 1.0 mg/mL rabbit anti-mouse IgG solution was sprayed on the NC membrane at a speed of 5 μL/cm via XYZ 3060 dispensing platform as T and C lines, respectively. The sample pad was initially treated with a treatment solution (10 mM PBS (pH 7.2), 1% Tetronic 1307 (S9) (w/v), 0.5% BSA (w/v), and 0.1% Tween-20 (v/v)) and incubated at 37°C for 4 h. The sample pad, NC membrane, and absorbent pad were then sequentially pasted on the PVC pad in an environment with a humidity of less than 35% to ensure the successful movement of the sample solution. Finally, the immunochromatographic strip was cut into a 0.4 cm width strip into the plastic shell and stored in a sealed aluminum foil bag at room temperature.

### 2.4 Detection procedure of the TRFM-LFIA strip

The TRFM-LFIA measurement procedure was performed as follows. First, the PdG standard solution or sample solution was mixed with a certain dilution of the TRFM-mAb conjugate in a centrifuge tube. After pre-incubation for 3 min, 70 μL mixed solution was added dropwise to the sample pad, and the results were observed under ultraviolet irradiation after 15 min. The fluorescence intensity of the T and C lines was determined by a fluorescent strip scanning reader for quantitative analysis.

### 2.5 Evaluation of the TRFM-LFIA strip

In addition to the optimization of reaction mode and system, reasonable performance evaluation is the basis of developing strip for practical application. The PdG standard solution was used to evaluate several important properties of TRFM-LFIA, such as sensitivity, stability, specificity, and precision (Li et al., 2021a). A series of concentrations of PdG standard solution in PBS buffer were analyzed by TRFM-LFIA to evaluate sensitivity. Then, the fluorescence intensity of T and C lines was recorded by a fluorescent strip scanning reader, and the calibration curve was established according to the fluorescence intensity ratio (T/C) of T and C lines and the concentration of PdG. The LOD was calculated based on the test results of 11 negative standard samples to obtain the sensitivity of the TRFM-LFIA. The same batch of immunochromatographic strips were stored for 1–4 weeks at 4°C 25°C, and 37°C in the dark to confirm the accuracy of sample analysis, and the fluorescence signal value T/C was recorded to evaluate stability. The fluorescence intensity of TRFM-mAb was determined under different conditions to evaluate its stability. Fluorescence detection was carried out on an F-2700 fluorescence spectrophotometer (Hitachi, Japan), with the excitation voltage was 700 V and the scanning speed 3,000 nm min^−1^. The excitation and emission slits were set to 5 nm, and the response time 0.4 s. The excitation wavelength was kept at 365 nm to record the emission spectrum of the solution with the wavelength range of 385 nm–700 nm. All the tests were performed in triplicate. Possible hormones in urine (i.e., E3 (5,000 ng/mL), LH (30 mIU/mL), and HCG (30 mIU/mL) were analyzed to determine the specificity of TRFM-LFIA for PdG detection. The same batch and different batches of TRFM-LFIA were used to detect PdG to evaluate the precision of the prepared immunochromatographic strips. Precision was assessed according to the intra-assay and inter-assay recoveries and coefficient of variation (CV). The data analysis was performed through Origin 2023b and Nano Measurer.

### 2.6 Real sample analysis

Concentrations of PdG in different urine samples were measured to evaluate the practicability of the prepared TRFM-LFIA strip. The urine samples were collected from two healthy adults without any pre-treatment. Different urine samples (urine sample 1 and urine sample 2) were spiked with 100–1,500 ng/mL PdG (final concentration). The spiked urine samples were subjected to fluorescence measurement according to the above procedure and the concentrations of PdG were calculated according to the standard curve. All the experiments were repeated three times, and the results were expressed as mean ± standard deviation (SD).

## 3 Results and discussion

### 3.1 Principle of TRFM-LFIA strip for detection of PdG in urine samples

The principle of quantitative detection of PdG by TRFM-LFIA test strip was shown in Scheme 1. Based on the immune response between antigen and antibody, the TRFM-LFIA test strip was constructed by the competition immunological method (Zhou et al., 2023). Firstly, the carboxyl group on the surface of TRFM activated by EDC and Sulfo-NHS was aminated with the amino group of anti-PDG-mAb to obtain TRFM-mAb as a fluorescent tracer. The time-resolved technique (Sun et al., 2021) was used to reduce the interference of the background fluorescence signal and improve the sensitivity of PdG quantitative detection. For negative samples, TRFM-mAb was captured by PdG-BSA to produce fluorescent response while they passed through PdG-BSA fixed on the T-line, and the remaining TRFM-mAb was captured by rabbit anti-mouse IgG on the C-line to produce fluorescent response. Eventually, two fluorescent bands of T-line and C-line were observed under ultraviolet light. In contrast, the PdG in the urine sample competed with the PDG-BSA conjugate to combine with TRFM-mAb to form complexes, and the complexes formed by TRFM-mAb and PdG in the positive sample moved forward along the cellulose nitrate membrane. The fluorescence response at the T-line was inversely proportional to the PdG concentration, and the fluorescence intensity of the T-line gradually decreased with the increase of PdG concentration in the sample. The concentration of PdG in the sample can be measured by detecting the fluorescence intensity at the T-line.

### 3.2 Preparation and characterization of TRFM and TRFM-mAb

Different PBS buffer pH and the ratio of anti-PdG-mAb to TRFM had different effects on the preparation of TRFM-mAb (Wang et al., 2014; Li et al., 2015). As shown in Supplementary Figure S1A, the T-line fluorescence intensity was stronger at pH 6.0 and 8.0 in the absence of PdG. On the other hand, the TRFM-LFIA strip constructed based on competitive immunoassay decreased the fluorescence intensity of T-line with the addition of analyte. And the T-line intensity change rate (T/T_0_) was the lowest at pH 8.0, indicating that the pH of 8.0 brought a greater change in T-line fluorescence intensity compared with other pH values. The charge of proteins depends on the number and type of ionizable amino acid groups. Monoclonal antibodies usually have a PI value greater than 8, comprised between 8.0 and 9.0 (Fekete et al., 2015; Goyon et al., 2017). This may be due to the fact that the pH was near the isoelectric point of anti-PdG-mAb, and the charge distribution of the antibody was uniform, which had a better coupling effect with TRFM (Jiang, et al., 2022). Therefore, the pH of 8.0 was selected as the optimal pH for the coupling reaction. As shown in Supplementary Figure S1B, the T-line fluorescence intensity initially increased with the increase of anti-PdG-mAb to TRFM ratio, and slightly decreased with the continuous increase of the ratio of anti-PdG-mAb to TRFM in the absence of PdG. The anti-PdG-mAb to TRFM ratio of 125 μg/mg had a higher effect on T-line fluorescence intensity than other ratios in the presence of PdG. Finally, a pH of 8.0 and an anti-PdG-mAb to TRFM ratio of 125 μg/mg were selected for the preparation of TRFM-mAb.

**FIGURE 1:**
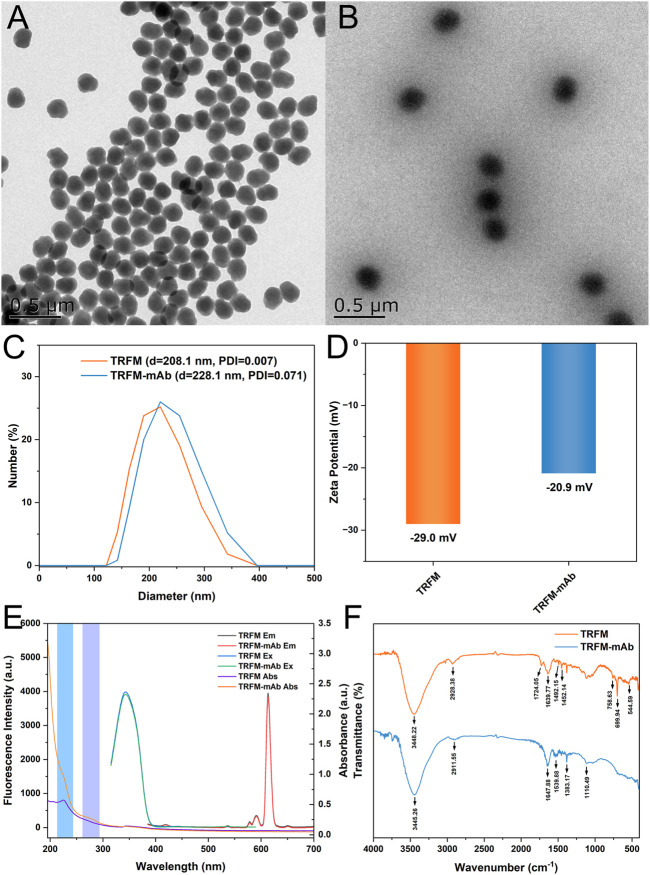
TEM images of TRFM **(A)** and TRFM-mAb **(B)**; the hydrated particle sizes of TRFM and TRFM-mAb **(C)**; the Zeta potentials of TRFM and TRFM-mAb **(D)**; the optical properties of TRFM and TRFM-mAb **(E)** and FT-IR spectra of TRFM and TRFM-mAb **(F)**.

The morphology and particle size of TRFM and TRFM-mAb were characterized by transmission electron microscopy (TEM, Figures 1A,B). TEM images showed that TRFM possessed a monodisperse spherical structure with an average diameter of approximately 202.6 nm. After coupling, the particle size of TRFM-mAb increased and irregular protrusions appeared on the surface due to the covalent binding of the antibody to the microsphere (Li et al., 2021a). This result demonstrated the successful coupling of anti-PdG-mAb to TRFM. Dynamic light scattering (DLS) results showed that the hydrodynamic diameter of TRFM was 208.1 nm and the dispersion index was 0.007. The TRFM-mAb had a hydrodynamic diameter of 228.1 with the dispersion index of 0.071 (Figure 1C). As shown in Figure 1D, the Zeta potential of TRFM-mAb (−29.0 mV) increased compared with that of TRFM (−20.9 mV), which was due to the amino group of anti-PdG-mAb was covalently bound to the carboxyl group on the surface of TRFM, and the carboxyl group on the surface of the TRFM was occupied, leading to an increased in Zeta potential (Chen et al., 2021). As shown in Figure 1E, the coupling also produced a new absorption peak at around 270 nm, which was speculated to be caused by TRFM coupling with the antibody (Chen et al., 2019). TRFM-mAb produced by anti-PdG-mAb labeled with TRFM caused no change in the optical properties of TRFM. When TRFM-mAb was excited at 365 nm, the maximum emission peak appeared at 610 nm. These results further confirmed the successful coupling of anti-PdG-mAb with TRFM. In addition, the TRFM was demonstrated to have excellent stability, and the corresponding results were shown in Supplementary Figure S2. The Fourier transform infrared spectroscopy (FT-IR) of TRFM and TRFM-mAb were illustrated in Figure 1F to investigate the functional groups. The absorption peaks of TRFM at 3448.22 cm^−1^ were attributed to the stretching vibration of O−H, and the characteristic peaks at 2928.38 cm^−1^ were attributed to the stretching vibration of saturated C–H on the benzene ring. The two absorption peaks at 1492.15 cm^−1^ and 1452.14 cm^−1^ were attributed to the bending vibrations of methylene groups. The two strong absorption peaks at 699.94 cm^−1^ and 758.63 cm^−1^ are monosubstituted characteristic peaks on the benzene ring. The characteristic peak of TRFM at 544.49 cm^−1^ was attributed to the vibration of the Eu−O bond. The weak absorption peak at 1724.05 cm−1 was the asymmetric stretching vibration peak of carbonyl C=O. For the TRFM-mAb, the absorption peaks at 3445.26 cm^−1^ were attributed to the stretching vibration of O−H and N−H, and the characteristic peaks at 2911.55 cm^−1^ belong to the stretching vibration peak of saturated C–H. The absorption peaks at 1647.88, 1383.17, and 1110.49 cm^−1^ were attributed to the stretching vibration of N−H, C–H, and C−N, respectively. The characteristic C=O stretching peak at 1539.88 cm^−1^ were attributed to the amide group (Lin et al., 2022; Yue et al., 2022; Zan et al., 2022; Xu, et al., 2023). It demonstrated that amide bond was formed between the TRFM and anti-PdG-mAb, which confirmed the successful coupling of TRFM and anti-PdG-mAb.

### 3.3 Determination of TRFM-LFIA strip detection parameters

#### 3.3.1 pH of the reaction solution

**FIGURE 2:**
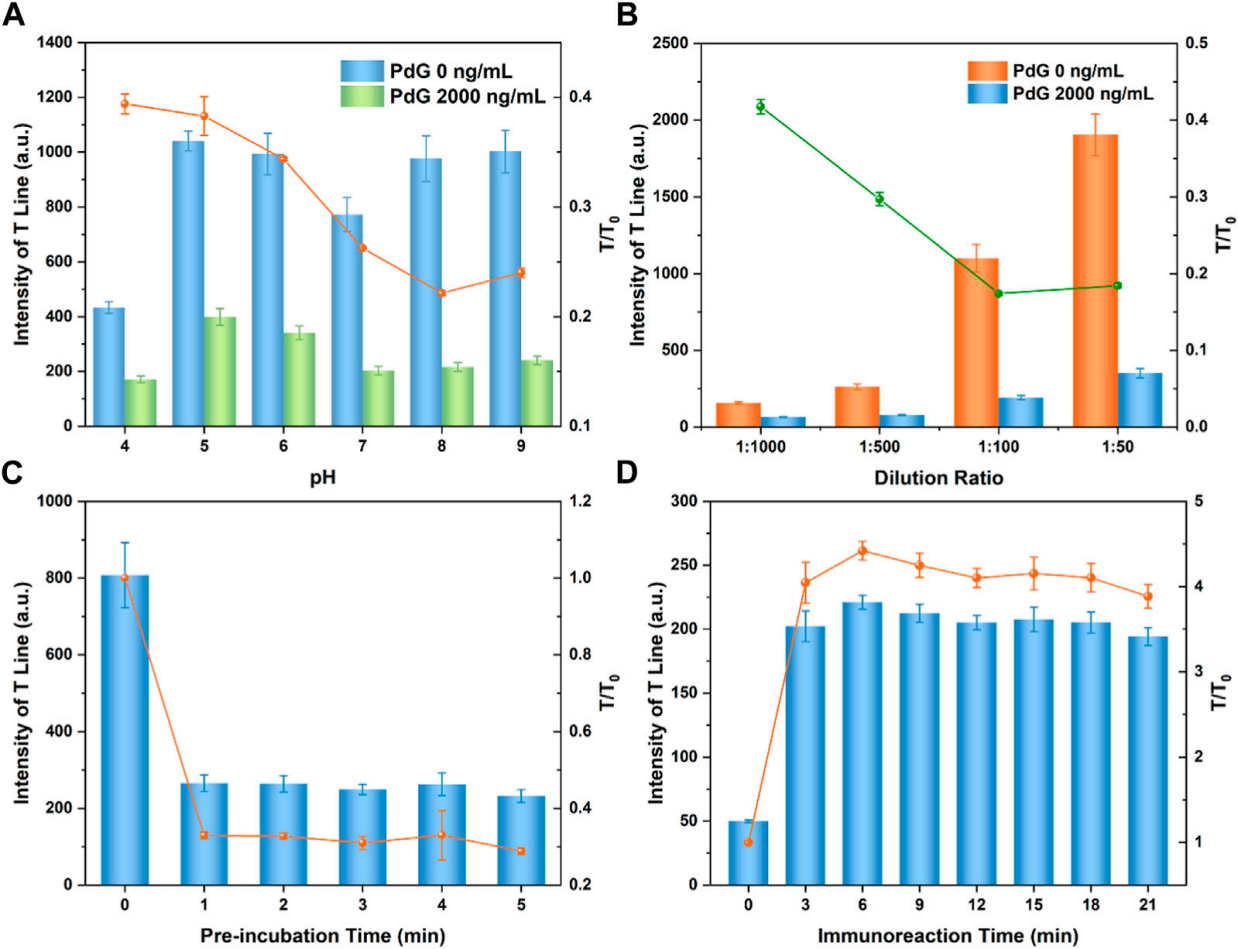
Effect of pH value of the reaction solution **(A)**; dilution ratio of TRFM-mAb **(B)**; pre-incubation **(C)** and immunoreaction time **(D)**.

The pH value of the reaction solution significantly affects the performance of the LFIA strip. As shown in Figure 2A, the pH of the reaction solution was optimized (Rodriguez-Cervantes et al., 2013; H; Zhang et al., 2019). When the pH value of the TRFM-mAb reaction system was ≥8 or ≤6, flocculation and precipitation occurred in acidic or alkaline environments (Li et al., 2021b). The fluorescence intensity at the T-line increased significantly, and the value of T/T_0_ gradually decreased with the increase of pH of the reaction solution; however, the extreme pH adversely affected T/T_0_. Considering the decrease of binding capacity of the reaction system under alkaline conditions, the reaction solution of pH 8.0 was determined to be the best pH value for detection.

#### 3.3.2 Dilution ratio of TRFM-mAb

The dilution ratio of the TRFM-mAb was optimized to improve the detection sensitivity (Wei et al., 2023). As shown in Figure 2B, the fluorescence intensity at the T-line of the negative control group decreased with the increase of the TRFM-mAb dilution ratio. However, when the dilution ratio of TRFM-mAb was 1:10, the background fluorescence of the strip was deeper than the test range (Supplementary Figure S3). When the dilution ratio of TRFM-mAb was in the range of 1:50 to 1:100, the fluorescence intensity at the T-line was higher, which was suitable for LFIA based on competitive mode (M. Li et al., 2021a). In contrast, the fluorescence intensity was relatively weak when the TRFM-mAb dilution ratio exceeded 1:500, failing to meet the requirement for the fluorescence quantitative detection of the target. Considering the above results, 1:100 was chosen as the optimal TRFM-mAb dilution ratio, which could also reduce raw material costs.

#### 3.3.3 Pre-incubation time of TRFM-mAb and PdG

In order to reduce the non-repeatability between different TRFM-LFIA strips due to the random diffusion of TRFM-mAb along the glass fiber, which may also reduce the sensitivity, the proposed TRFM-LFIA strip eliminated the traditional conjugate pad (Molinelli et al., 2009; Liu et al., 2023). Therefore, TRFM-mAb was pre-mixed and pre-incubated with a standard solution containing PdG prior to detection. The pre-incubation time was optimized in Figure 2C, and the fluorescence intensity at the T-line and the ratio of T/T_0_ gradually decreased with the increase of pre-incubation time and reached stability after 1 min. The specific binding reaction of TRFM-mAb and PdG was completed due to the increase of pre-incubation time. Considering the above results, 3 min was chosen as the optimal pre-incubation time.

#### 3.3.4 Immunoreaction time for TRFM-LFIA strip

The immunoreaction was a dynamic process, and the fluorescence intensity of the T-line and C-line changed dynamically with the increase of reaction time (Sun, et al., 2021; Su et al., 2022). The immunoreaction dynamics of TRFM-LFIA for PdG detection was shown in Figure 2D, the fluorescence intensity at the T-line and the T/T_0_ ratio gradually increased with the extension of the immunoreaction time, and gradually stabilized after 15 min. In this case, the optimal immunoreaction time was determined to be 15 min.

### 3.4 Sensitivity of TRFM-LFIA strip

**FIGURE 3:**
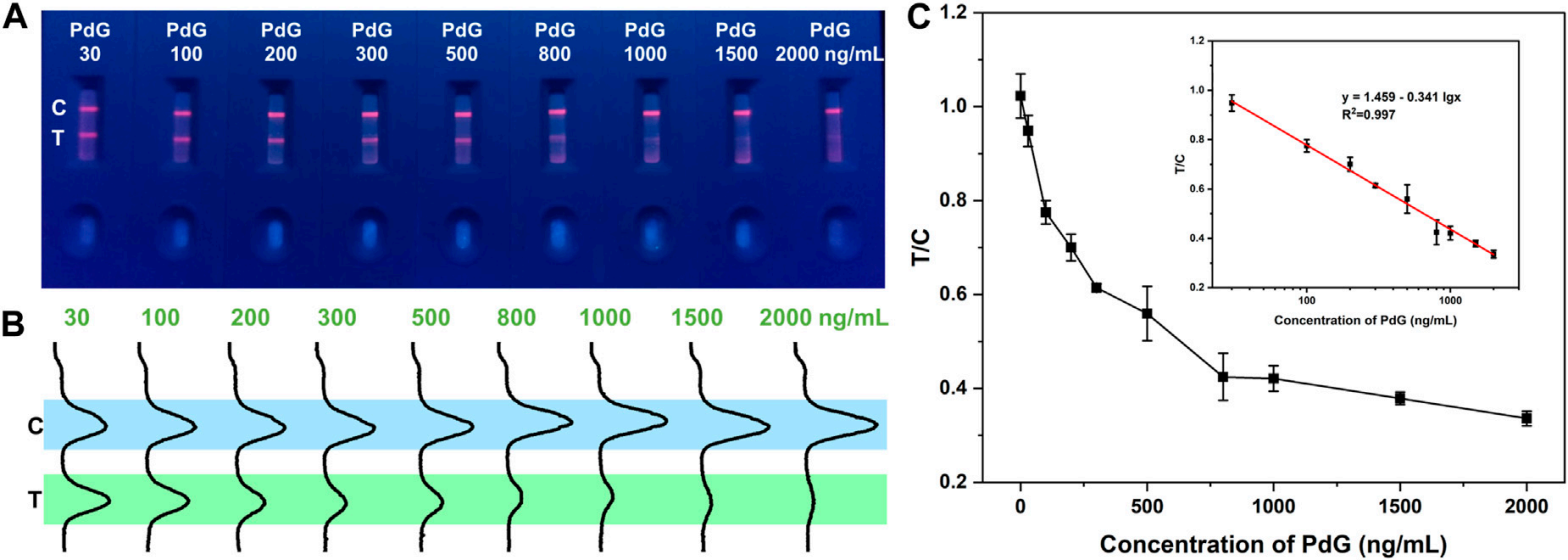
Detection results of TRFM-LFIA strip loaded with different concentrations of PdG. **(A)** Image of the detection results of TRFM-LFIA strip under ultraviolet light; **(B)** fluorescence intensity results of the TRFM-LFIA strip; **(C)** the relationship between T/C ratio and PdG concentration (Inset: the standard curves of TRFM-LFIA strip analysis for PdG).

Different concentrations of PdG were detected to evaluate the sensitivity of the TRFM-LFIA strip under the optimal detection parameters. As shown in Figure 3A, bright strips were observed at line C of all test strips under ultraviolet light, indicating that the TRFM-LFIA strips worked well. As shown in Figure 3B, the fluorescence intensity at the T-line gradually decreased with the increase of PdG concentration, which conformed to the detection principle based on competition mode. The fluorescence intensity of the T and C lines of the TRFM-LFIA strip at different concentrations of PdG was read by a fluorescent strip scanning reader. The competitive inhibition curve was drawn with PdG concentration as the horizontal coordinate and T/C value as the vertical coordinate (Figure 3C). The fitting curve accorded with the principle of immunoassay and had a high correlation, which better reflected the relationship between PdG concentration and the ratio of T/C. The corresponding fitting equation was as follows: *T/C* = 1.459–0.341 lg *C*_*PdG*_ (R^2^ = 0.997), where *T* and *C* respectively represent the fluorescence intensity of the T line and C line, and *C*_*PdG*_ represents the concentration of PdG. The dynamic range was 30–2,000 ng/mL, and the detection limit of TRFM-LFIA was 8.39 ng/mL (LOD = 3*σ/k*), where *σ* represents the standard deviation of 11 negative standard samples, and *k* represents the slope of the standard curve. The corresponding calculation process was shown in Supplementary Table S1. The limit of quantification (LOQ) can be calculated by the following equation: LOQ = 10*σ/k*, and the calculated LOQ was 27.97 ng/mL, where *σ* represents the standard deviation of the y-intercept of the regression line and *k* represents the slope of the standard curve. Therefore, we determined the lower dynamic range to be 30 ng/mL. And there was a very slight fluorescence signal of the T-line at the PdG concentration of 2,000 ng/mL, which cannot be accurately recorded when the PdG concentration was higher. Therefore, the detection range was 30–2,000 ng/mL. Compared with other detection methods, the TRFM-LFIA strip proposed in this study had a higher sensitivity for the detection of PdG, and the detection method was simple and fast.

Compared with the previous reports of PdG detection in urine samples, the TRFM-LFIA strip was convenient to use and less dependent on the instrument (Supplementary Table S2). No large instruments were required, only a low-cost UV lamp was needed to read the test results for qualitative detection. The current colloidal gold strips mostly detect ovulation by setting a detection threshold (usually 5 μg/mL). The method developed in this paper achieved quantitative detection by using a fluorescent strip scanning reader, and the LOD of the TRFM-LFIA strip was also lower than that of the colloidal gold test strip. In addition, the TRFM-LFIA strip also had the advantage of low cost. These advantages indicated that the TRFM-LFIA strip developed in this work exhibited enormous potential for PdG self-test.

### 3.5 Stability and specificity of TRFM-LFIA strip

**FIGURE 4:**
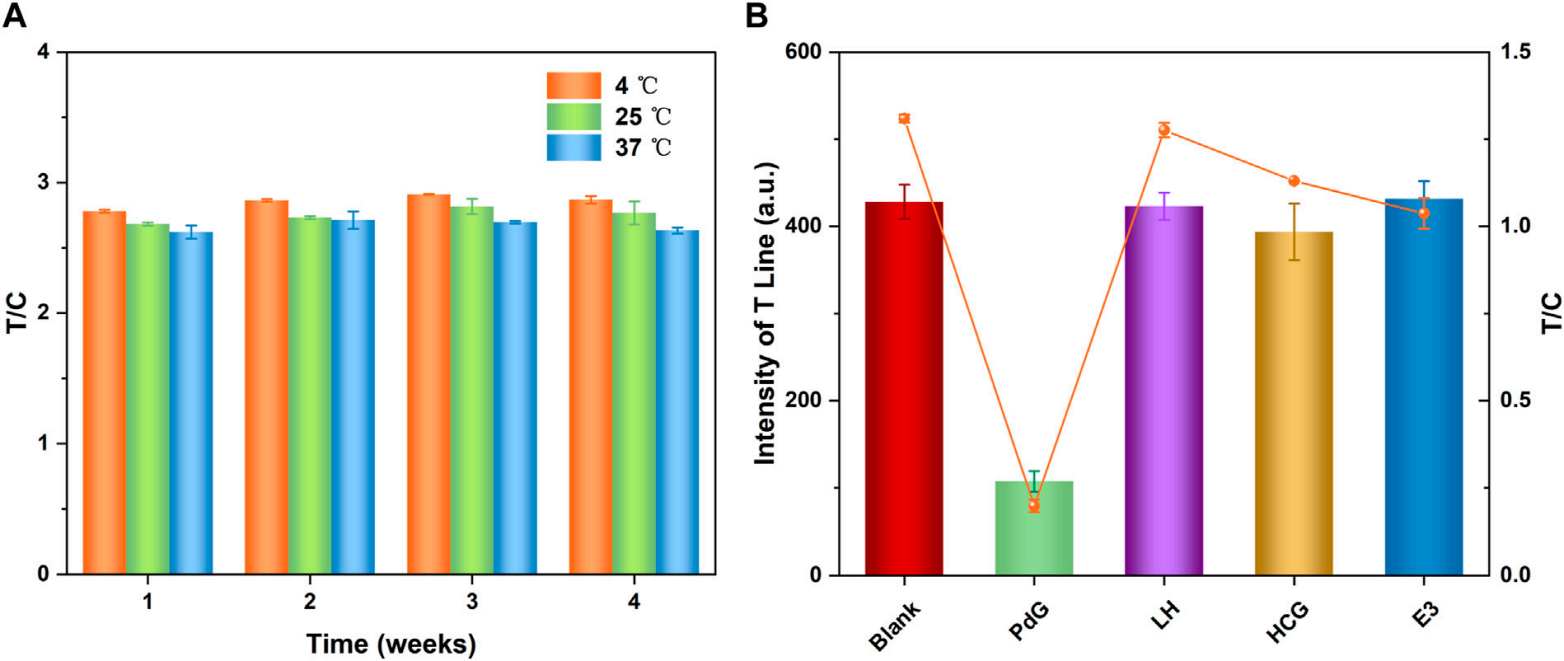
Detection stability of the TRFM-LFIA strip preserved with different temperatures and periods **(A)** and response of fluorescence signal (T-line intensity) of TRFM-LFIA strip for PdG, LH, HCG and E3 **(B)**, the concentration of PdG and E3 was 5,000 ng/mL, the concentration of LH and HCG was 30 mIU/mL.

The practical applicability of TRFM-LIFA was determined by the stability of the immunochromatographic strip. The stability results (Figure 4A) showed that the fluorescence intensity of TRFM-LFIA strips stored at 4°C, 25°C, and 37°C for 1–4 weeks was similar. According to the Arrhenius equation, the stability of the test strip stored at 4°C for 1 year is basically similar to that stored at 37°C for 1 month (Wang et al., 2009; Liu et al., 2021), indicating that the TRFM-LFIA strip prepared in this study had good stability and met the commercial conditions. In addition, the TRFM-mAb was demonstrated to have excellent stability under different conditions, and the corresponding results were shown in Supplementary Figure S4. The potential interference of several hormones (LH, HCG, E3) that may be present in urine was investigated in order to evaluate the specificity of the TRFM-LFIA strip in this experiment. As shown in Figure 4B, the T-line fluorescence intensity and T/C value of the PdG immunochromatographic strip were significantly lower than those of other hormones at the same concentration, indicating that the TRFM-LFIA strip showed excellent specificity and was suitable for the specific detection of PdG in actual urine samples.

### 3.6 Precision of TRFM-LFIA strip

The precision of the TRFM-LFIA strip proposed in this study was evaluated by selecting the same or different batches of strips to detect positive samples. As shown in Table 1, the recovery of the same batch of TRFM-LFIA strip was 102.75–105.31%, and the CV was 1.24–3.70%; the recovery of different batches of TRFM-LFIA strip was 92.56–96.12%, and the CV was 0.71–6.68%. The above analysis results indicated that the TRFM-LIFA proposed in this study exhibited high precision and could meet the quantitative detection of PdG.

**TABLE 1:** The precision of the TRFM-LFIA strip in PdG-spiked detection. (*n* = 3).

PdG concentration (ng/mL)	Intra-assay			Inter-assay		
	Mean	Recovery (%)	CV (%)	Mean	Recovery (%)	CV (%)
250	263.27	105.31	3.70	240.30	96.12	2.37
500	513.77	102.75	3.22	462.82	92.56	0.71
1000	1049.76	104.98	1.24	950.38	95.04	6.68

### 3.7 Analysis of spiked PdG urine samples

PdG is mainly present in the urine as a progesterone metabolite. To evaluate the applicability of the TRFM-LFIA strip, the strip was applied to the detection of PdG in two urine samples. The standard addition recovery experiment was conducted by adding standard concentration PdG to the negative urine sample, and the results were shown in Table 2. The recoveries of PdG in urine samples ranged from 97.39% to 112.64%, and the RSDs were less than 8.56% (*n* = 3). The results indicated that the TRFM-LFIA showed comparative recoveries, which was practically feasible for rapid detection of PdG in the urine samples.

**TABLE 2:** Applications of TRFM-LFIA strip in PdG determination in real urine samples (*n* = 3).

Sample	PdG added (ng/mL)	PdG found (ng/mL)	Recovery (%)	RSD (%)
Urine 1	100	103.56	103.56	1.24
	800	812.60	101.57	2.32
	1500	1460.90	97.39	8.56
Urine 2	100	104.44	104.44	3.55
	800	851.84	106.48	3.49
	1500	1689.54	112.64	2.08

## 4 Conclusion

In summary, we have successfully fabricated a time-resolved fluorescence microsphere-lateral flow immunochromatography strip for quantitative detection of PdG. By successful coupling of TRFM with anti-PdG-mAb, the designed TRFM-LFIA assay exhibited wonderful sensitivity and selectivity response to PdG. And over the range of 30–2,000 ng/mL of PdG, the fluorescence intensity ratio of T/C showed a good linear relationship with an R^2^ of 0.997. The corresponding LOD was calculated as low as 8.39 ng/mL. Most importantly, the designed TRFM-LFIA strip could rapidly respond to PdG in urine samples with good sensitivity, selectivity, and stability, revealing the excellent feasibility and great potential application in the rapid detection of metabolites.

## Data Availability

The original contributions presented in the study are included in the article/Supplementary Material, further inquiries can be directed to the corresponding authors.
