# Supplementary material for: A time-resolved fluorescence microsphere-lateral flow immunochromatographic strip for quantitative detection of Pregnanediol-3-glucuronide in urine samples
Source: Front Bioeng Biotechnol. 2023 Dec 19;11:1308725. doi: 10.3389/fbioe.2023.1308725 (PMC10758493; doi:10.3389/fbioe.2023.1308725)
Supplement: Supplementary file 1 [file DataSheet1.PDF]

Supplementary Material

A time-resolved fluorescence microsphere-lateral flow immunochromatographic strip for quantitative detection of Pregnanediol-3-glucuronide in urine samples

Jiasheng Lin[1,2]‡, Sanhua Li[3]‡, Benchen Ye[2,4], Weigang Zheng[2,4], Huihui Wang[2,4], Ying Liu[1,2], Dong Wang[1,2], Zaihui Wu[1,2], Wen-fei Dong[1,2]*, Minghui Zan[1,2,4]*

[1]CAS Key Laboratory of Biomedical Diagnostics, Suzhou Institute of Biomedical Engineering and Technology, Chinese Academy of Science (CAS), Suzhou 215163, P. R. China;

[2]Zhengzhou Institute of Biomedical Engineering and Technology, Zhengzhou, Henan 450001, China;

[3]Henan Province Joint International Laboratory for Bioconjugation and Antibody Coupling, Zhengzhou, Henan 450001, China;

[4]Zhongke Technology Achievement Transfer and Transformation Center of Henan Province, Zhengzhou, Henan 450001, China;

‡ These authors contributed equally to this work;

*** Correspondence:**
Corresponding Author
wenfeidong@sibet.ac.cn (Wen-Fei Dong), zanmh@sibet.ac.cn (Minghui Zan)

Materials and methods

Chemicals and reagents

Pregnanediol-3-glucuronide (PdG) was purchased from Kehaojia Biotechnology Co., Ltd. (Wuhan, China). Carboxylate-modified polystyrene time-resolved fluorescent microspheres (TRFM) (200 nm, red fluorescence [365/610 nm, λex/λem, 1% solid]) were provided by Xian Qiyue Biotechnology Co., Ltd. (Xian, China). PdG-BSA and the mouse monoclonal antibodies against PdG (anti-PdG-mAb) were purchased from Clongene Biotech Co., Ltd. (Hangzhou, China). The Rabbit anti-mouse immunoglobulin (IgG) as the secondary antibody, Luteinizing Hormone (LH), Human Chorionic Gonadotropin (HCG), and Potassium carbonate (K_2CO_3) were purchased from Sangon Biotech Co., Ltd. (Shanghai, China). Estriol (E3) was purchased from LGC Science Ltd. (Nanjing, China). Proclin 300 was purchased from Bioss Biotechnology Co., Ltd. (Beijing, China). N-(3-Dimethylaminopropyl)-N'-ethylcarbodiimide hydrochloride (EDC), N-Hydroxysulfosuccinimide sodium salt (Sulfo-NHS), Bovine serum albumin (BSA), D-(+)-Trehalose Anhydrous and Tween-20 were purchased from Aladdin (Shanghai, China). PBS buffer, sucrose, and Tetronic 1307 (S9) were purchased from Adamas (Shanghai, China). MES buffer was purchased from SenBeiJia Biological Technology Co., Ltd. (Nanjing, China). Sodium chloride (NaCl), Disodium hydrogen phosphate (Na_2HPO_4), and Sodium dihydrogen phosphate (NaH_2PO_4) were purchased from Fuchen Chemical

Reagent Co., Ltd. (Tianjin, China). Ethanol absolute was purchased from Fuyu Fine Chemical Co., Ltd. (Tianjin, China). Sample pad (FH-8975), absorbent pad (FH-S073), and nitrocellulose (NC) membrane (CN140) were purchased from Fenghang Technology Co., Ltd. (Hangzhou, China). All the other reagents in the present work were analytical grade without any purification. Deionized water used throughout the experiments was acquired by the laboratory purification system.

Apparatus

Transmission electron microscopy (TEM) images were recorded with a Tecnai 12 Transmission Electron Microscope (FEI, USA). Fourier transform infrared (FT-IR) spectra were recorded by Fisher Nicolet iS5 spectrometer (Thermo, USA). Ultraviolet-visible (UV-vis) absorption spectra were recorded by a Shimadzu UV-2550 spectrophotometer (Shimadzu, Japan). Fluorescence detection was carried out on an F-2700 fluorescence spectrophotometer (Hitachi, Japan). Zeta potential and hydration particle size were measured by a Zetasizer Nano ZS90 instrument (Malvern, UK). The strip scanning results were recorded by the Suzhou Hemai Fluorescent Strip Scanning Reader (Hemai, China). The test strips were prepared by XYZ 3060 dispensing platform (Bio Dot, USA). The test strips were cut through the ZQ 2002 automatic test strip cutter (Jinbiao, China).

Results and discussion

The conditions of the coupling reaction process were optimized in **Figure S1**, and the optimal coupling conditions were pH 8.0 and the ratio of anti-PdG-mAb to TRFM 125 μg/mg.

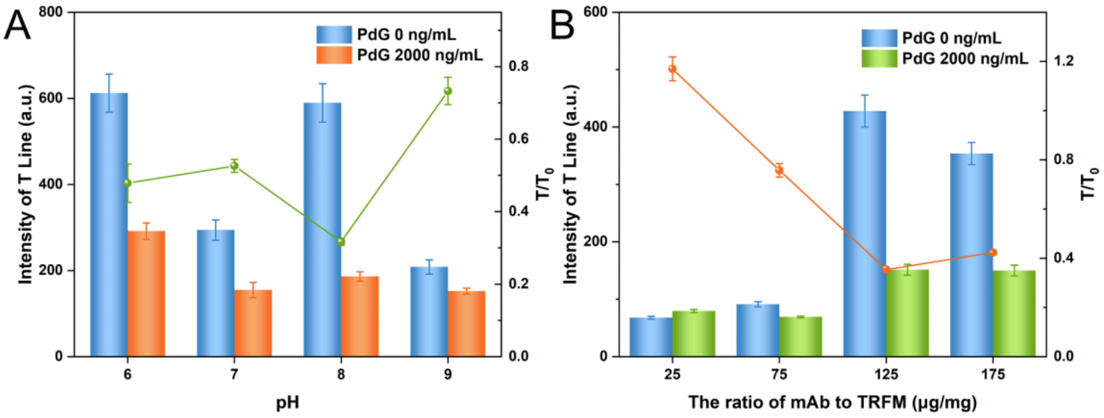

Figure S1 Optimization of preparation conditions of TRFM-mAb (A) the pH of the buffer and (B) the ratio of anti-PdG-mAb to TRFM.

As shown in **Figure S2A**, the fluorescence intensity of TRFM remained relatively stable under different ionic strengths (NaCl, 0.1 to 1.0 mol/L). The stability of TRFM under high ionic strength conditions enabled TRFM to be used in extreme environments. As shown in **Figure S2B**, the fluorescence intensity of TRFM decreased slightly under pH 11.0, but exhibited stable fluorescence intensity in a wide range of pH (3.0−10.0). Moreover, TRFM exhibited a relatively stable fluorescence intensity after long-term UV irradiation (**Figure S2C**). This result showed that TRFM had a high stability, which was conducive to their application in chemical and biological analysis.

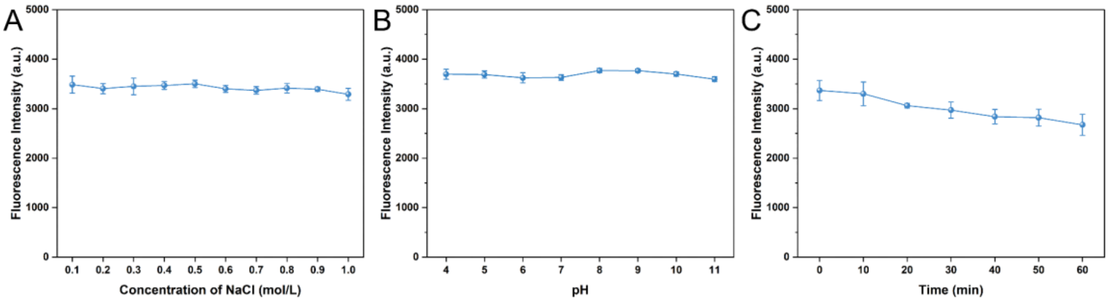

Figure S2 Effect of different ionic strength (A); pH (B); irradiation time with a 365 nm UV-lamp (C) on the fluorescence intensity of the TRFM.

Figure S3 showed the detection image of the TRFM-LFIA strip at different dilution ratios of TRFM-mAb. The fluorescence intensity of the TRFM-LFIA strip was increased with the dilution ratio decreasing. However, the background fluorescence intensity of the TRFM-LFIA strip was stronger when the dilution ratio was 1:10. When the dilution ratio ranged from 1:50 to 1:100, the fluorescence intensity at the T-line was higher, which was suitable for the competitive mode. Considering the above results, 1:100 was chosen as the optimal dilution ratio for detection.

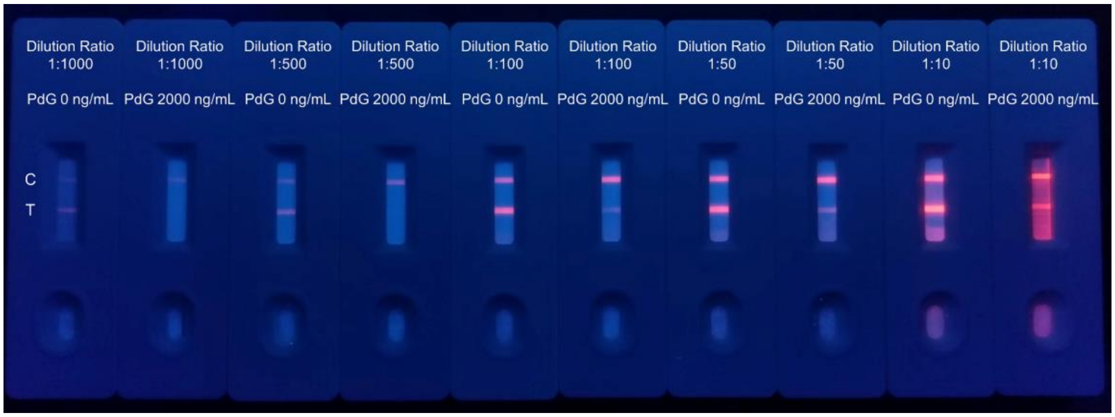

Figure S3 The detection image of TRFM-LFIA strip under ultraviolet irradiation in different TRFM-mAb dilution ratios.

We tested the T/C of 11 blank samples and calculated the standard deviation (σ) to be 0.105. According to the detection limit equation LOD = $3\sigma/k$ (where σ was 0.105 and k was 0341), the lg C_{PdG} was 0.924, and the detection limit of TRFM-LFIA was 8.39 ng/mL.

Table S1 the corresponding data table of LOD.

σ	k	Lg $C=3\sigma/k$	C (ng/mL)
0.105	0.341	0.924	8.39

Compared with the previous reports of PdG detection in urine samples, the TRFM-LFIA strip was convenient to use and less dependent on the instrument (Table S2).

Table S2 Comparison of our assay and previous reports of PdG detection in urine.

Methodology	LOD	Linear range	Cost	Accessibility	Readout system	Reference
LC–MS/MS	0.01 ng/mL	0.38-100 ng/mL	High	Low (need laboratory)	Instrument	(Chen et al., 2020)
Radioimmunoassay	-	-	High	Low (need laboratory)	Instrument	(Mendizabal, et al., 1984)
Immunochemical Assay	0.4 ng/mL	0-50 ng/mL	Moderate	Low (need laboratory)	Instrument	(Hiroi et al., 1986)
Enzyme immunoassays	-	-	High	Low (need laboratory)	Instrument	(O'Connor et al., 2003)
Colloidal gold strip	-	-	Low	High (OTC)	Eyes	(Leiva, et al., 2019)
TRFM-LFIA	8.39 ng/mL	30−2000 ng/mL	Low	High (OTC)	Eyes/Instrument	This work

As shown in **Figure S4A**, the fluorescence intensity of TRFM-mAb remained stable over a wide pH range (4.0−12.0). As shown in **Figure S4B**, the fluorescence intensity of TRFM-mab remained stable at different ion concentrations (NaCl, 0.1 to 1.0 mol/L), and its stability under high ion strength conditions ensured that TRFM-mAb was applied to actual urine samples. Moreover, TRFM-mAb exhibited relatively stable fluorescence intensity when stored at different temperatures for a week. The results showed that TRFM-mAb had high stability, which was conducive to its application in the detection of actual urine samples.

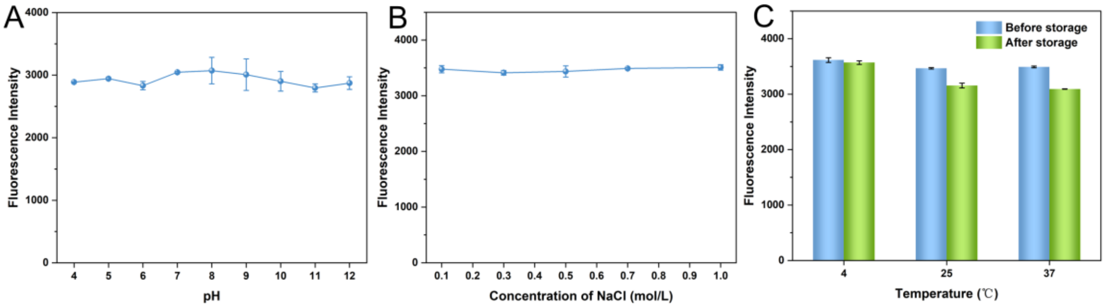

Figure S4 Effect of different pH (A); ionic strength (B); temperature (C) on the fluorescence intensity of the TRFM-mAb.

References

Chen, J., Hou, H., Chen, H., Luo, Y., He, Y., Wang, H., & Hu, Q. (2020). Quantitation of serum pregnanediol-3-glucuronide level in different endocrine statuses by LC-MS/MS. *J. Pharm. Biomed. Anal.* 184. doi: 10.1016/j.jpba.2020.113171

Hiroi, M., Kawagoe, S., Saito, H., Saito, N., Kubota, N., & Tsubota, N. (1986). Rapid immunochemical assay of pregnanediol-3-glucuronide in urine and its clinical application. *Gynecol. Obstet. Invest.* 22(4), 186-193. doi: 10.1159/000298912

Leiva, R., McNamara-Kilian, M., Niezgoda, H., Ecochard, R., & Bouchard, T. (2019). Pilot observational prospective cohort study on the use of a novel home-based urinary pregnanediol 3-glucuronide (PDG) test to confirm ovulation when used as adjunct to fertility awareness methods (FAMs) stage 1. *BMJ Open.* 9(5). doi: 10.1136/bmjopen-2018-028496

Mendizabal, A. F., Quiroga, S., Farinati, Z., Lahoz, M., & Nagle, C. (1984). Hormonal monitoring of early pregnancy by a direct radioimmunoassay of steroid glucuronides in first morning urine. *Fertil. Steril.* 42(5), 737-740.

O'Connor, K. A., Brindle, E., Holman, D. J., Klein, N. A., Soules, M. R., Campbell, K. L., & Wood, J. W. (2003). Urinary estrone conjugate and pregnanediol 3-glucuronide enzyme immunoassays for population research. *Clin. Chem.* 49(7), 1139-1148. doi: 10.1373/49.7.1139
